# Effects of the Ketogenic Diet in the Treatment of Gliomas: A Systematic Review

**DOI:** 10.3390/nu14051007

**Published:** 2022-02-27

**Authors:** Beatriz Sargaço, Patrícia Almeida Oliveira, Maria Luz Antunes, Ana Catarina Moreira

**Affiliations:** 1 Faculdade de Medicina, Universidade de Lisboa, 1649-028 Lisboa, Portugal; 2 ESTeSL-Escola Superior de Tecnologia da Saúde de Lisboa, Instituto Politécnico de Lisboa, 1990-096 Lisboa, Portugal; patricia.aao@gmail.com (P.A.O.); mluz.antunes@estesl.ipl.pt (M.L.A.); ana.moreira@estesl.ipl.pt (A.C.M.); 3 APPsyCI–Applied Psychology Research Center Capabilities & Inclusion, ISPA–Instituto Universitário, 1149-041 Lisboa, Portugal; 4 H&TRC-Health & Technology Research Center, 1990-096 Lisboa, Portugal

**Keywords:** ketogenic diet, glioma, glioblastoma, survival

## Abstract

The ketogenic diet (KD) is a restrictive therapeutic diet, distinguished by being hyperlipidic, normoproteic, and hypoglucidic. This diet simulates biochemical changes related to fasting periods to achieve systemic ketosis. The metabolic particularities of glioma tumors motivated the rise in investigations and nutritional strategies, such as KD, to modulate the glycemic response as a treatment. This systematic review followed the PRISMA recommendations and was published in PROSPERO, with the identification CRD42021264173. The databases used were EMBASE, PubMed/Medline, Scopus, and Web of Science, and the studies were analyzed using the web-based application Rayyan. To analyze the risk of bias, Cochrane RevMan 5 software was used. For the analysis and treatment of statistical data, Microsoft® Excel® was used. A total of nine original articles were included. Data on survival, symptomology, and quality of life were collected. Mean overall survival was 15.9 months. Constipation and fatigue were the most reported symptoms. In 44.4% of the studies, an improvement in the quality of life was found. The KD is supported by most published studies as an effective therapy in the treatment of malignant gliomas due to its positive effects on patient survival. It was not possible to conclude the effectiveness of KD on quality of life.

## 1. Introduction

The ketogenic diet (KD) is a restrictive therapeutic diet distinguished by being high-fat, normoproteic, and hypoglucidic, and is considered a non-pharmacological therapeutic method [1,2,3]. There are various types of KD according to the proportion of macronutrients. The four major types of KD are the Classic Ketogenic Diet (CKD), the Ketogenic Diet with Medium-Chain Triglycerides (MCT), the Modified Atkins Diet (MAD), and the Low Glycemic Index Diet (LGID). The classical type is classified by a ratio of 3:1 to 4:1, that is, 3 to 4 grams of lipids for every 1 gram of carbohydrate and protein [4,5]. The utilization of MCT as an energy source is due to the greater production of ketone bodies compared to long-chain fatty acids.

Generally, the KD provides a high amount of lipids (60–90% of total energy value (TEV)), a low carbohydrate content (4–19% TEV), and is adequate in protein, minerals, and vitamins [6,7].

All of these diets have lipid restrictions, but in the CKD, the restriction is more severe.

Metabolically, carbohydrates break down into glucose. However, in the absence of this energy source, the liver converts fat reserves into fatty acids and ketone bodies, allowing the utilization of these as alternative sources of energy. Ketone bodies, such as acetone, acetoacetate, and ß-hydroxybutyrate, overcome the blood–brain barrier and replace glucose usage [1,8,9].

KD simulates fasting periods by increasing ketones and lowering blood glucose, which leads to increased oxidation of fatty acids and production of acetyl-CoA [10,11].

When acetyl-CoA production exceeds the capacity that can be used on the tricarboxylic acid cycle, there is an increase in the production of ketone bodies, particularly β-hydroxybutyrate and acetoacetate, that will be used as energy sources in the brain [1,8,11,12]. 

Gliomas are primary, heterogeneous, invasive, and aggressive malignancies, which encompass most central nervous system tumors [1,5,13]. They originate from glial cells or stem cells, and after neoplastic transformation, acquire glial cell characteristics [14].

The survival prognosis for patients with malignant gliomas is around 12 to 15 months, with a survival rate of 5 years below 5% [13,15,16,17]. Thus, it is of high importance to develop new therapeutic strategies for these patients, especially those that can improve quality of life and/or survival [18,19].

The most frequent types of gliomas are glioblastomas, around 60–70% of all diagnosed cases, followed by anaplastic astrocytomas with 10–15% of cases and, finally, anaplastic oligodendrogliomas and anaplastic oligoastrocytomas with 10% of cases. The remainder includes gliomas such as anaplastic ependymomas and anaplastic gangliogliomas [20,21].

The metabolic particularities of cancer have motivated increased research on nutritional strategies to modulate the glycemic response during treatment. These nutritional strategies include the use of diets with high lipidic content and low carbohydrate content (such as KD), energy-restricted diets, or intermittent fasting [22]. The purpose of these diets is to induce a state of systemic ketosis to compensate for the decrease in glucose, originated from the lack of substrate [19].

In tumors, there are changes in the metabolism of cancer cells. These changes can be explained by the "Warburg effect", also known as aerobic glycolysis [18]. Otto Warburg, in 1924, observed that tumors had a metabolic phenotype with high rates of aerobic glycolysis [3].

Tumor cells depend on mitochondrial oxidative phosphorylation to generate adenosine triphosphate (ATP). Thus, when exposed to hypoxia, they switch to the less favorable anaerobic pathway of glycolysis. However, there are cancer cells that survive and proliferate generating ATP via glycolysis instead of oxidative phosphorylation, even in the presence of oxygen [1,8,15,18].

Cancer cells differ from other cells in their inability to use ketones as metabolic fuel. Tumor cells are very glucose-dependent for their growth and survival, while the remaining cells have the flexibility to alter their energy source from glucose to ketone bodies. With KD, tumor cells would no longer have an energy source for their growth [1,8,15,18].

By reducing glucose availability and providing ketone bodies as an alternative energy source, KD can offer a therapeutic approach directing the Warburg effect on glycolytic tumors such as malignant gliomas. Nutritional strategies aimed at glycemic modulation to explore tumor cells’ dependence on glucose have not yet been fully studied and investigated, so the existing clinical data are limited [19,22].

There have been several studies with the inclusion of this type of diet in the therapeutic intervention of diagnosed gliomas. Although systematic reviews addressing the impact of KD on the survival of patients with gliomas have been published in the past, this area of research has been intensively studied further, with some relevant original studies published since the latest review. The systematic analysis of the effect of this nutritional intervention covering the different types of diets, side effects, and quality of life is not yet well established. KD implementation raises the risk of many side effects, so it is essential to analyze this strand and all relevant studies published to date in a systematic review.

## 2. Materials and Methods

This systematic review followed the recommendations of PRISMA (Preferred Reporting Items for Systematic Reviews and Metanalyses), which is intended for systematic reviews and meta-analyses of clinical intervention studies [23]. This systematic review was published on the platform PROSPERO, the International Prospective Register of Systematic Reviews [24], on 17 July 2021 with the registration number CRD42021264173.

The used databases were EMBASE, PubMed/Medline, Scopus, and Web of Science, with the following keyword strategies:

- [(glioma) OR (glial cell tumor) OR (mixed glioma) OR (malignant glioma) OR (glioblastoma) OR (astrocytoma, grade IV) OR (glioblastoma multiforme) OR (giant cell glioblastoma) OR (high-grade glioma) OR (neoplastic stem cell) OR (cancer stem cells) OR (Warburg effect, oncologic) OR (astrocytoma) OR (brain tumor) OR (brain neoplasms) OR (glial cell) OR (glial precursors) OR (astrocytic tumor) OR (oligoastrocytic tumor) OR (neuro-glial tumor)] AND [(ketogenic diet) OR (low carbohydrate diet) OR (glucose) OR (glycolysis) OR (ketone bodies) OR (low carbohydrate) OR (diet therapy) OR (ketosis) OR (caloric restriction) OR (therapeutic ketosis) OR (low carb diet) OR (metabolic therapy) OR (adjuvant therapy) OR (energetic restriction) OR (energy restriction) OR (high-fat diet) OR (low carbohydrate high-fat diet) OR (keto-induction) OR (ketotherapy) OR (glucose restriction) OR (carbohydrate-restriction) OR (low carb) OR (hyper lipidic diet) OR (high-fat diet) OR (dietary intervention)].

The research included original articles published between January 2005 and May 2021 that included KD as a therapeutic intervention for the treatment of gliomas. After removing duplicate papers, the article selection was carried out according to titles, abstracts, and full texts. Case-control studies, retrospective and prospective cohort studies, and randomized clinical trials were included. The web-based application Rayyan, a software for systematic reviews and meta-analyses to facilitate initial screening through titles and abstracts, was used to analyze the identified studies [25]. This analysis for the identification of relevant publications was practiced by two reviewers who examined the articles independently for their inclusion, not knowing each other’s decisions. All divergences were resolved by consensus, and the divergences that did not reach consensus were resolved by a third reviewer.

For the analysis of the risk of bias, software designed for the development of systematic reviews and meta-analyses, Cochrane RevMan 5, was used. All the studies obtained were evaluated in depth by two reviewers regarding the risk of various biases to evaluate the quality of evidence. The risk of bias was classified as high risk, low risk, uncertain, or unapplicable risk [26].

Information was collected on the type of study (controlled or uncontrolled), the study design (prospective or retrospective), number of patients, age of patients, type of investigated glioma, stage of the disease, type of interventions performed, follow-up time, and type and duration of KD.

An initial survey was carried out on 24 April 2021 and repeated on 7 June 2021 to verify that all recent studies were covered, including the latest ones.

Microsoft® Excel® for Mac, version 16.41 (Microsoft Company), was used for the analysis and processing of statistical data.

### Inclusion and Exclusion Criteria

For this systematic review, inclusion criteria were considered:

- Original studies, published between 1 January 2005 and 30 May 2021;
- Clinical studies;
- Studies evaluating patients with malignant gliomas;
- Studies using the KD as an intervention/treatment for malignant gliomas;
- Studies published in English or Portuguese;
- Studies including patients of all ages, genders, and ethnicities in any country.

For the exclusion criteria, we considered:

- Articles such as comments, letters to the editor, reviews, conference editorials, opinion articles, or case reports;
- Articles associating KD with non-conventional therapies (e.g., intermittent fasting or perillyl alcohol);
- Studies that did not include results;
- Animal studies.

## 3. Results

**Figure 1:**
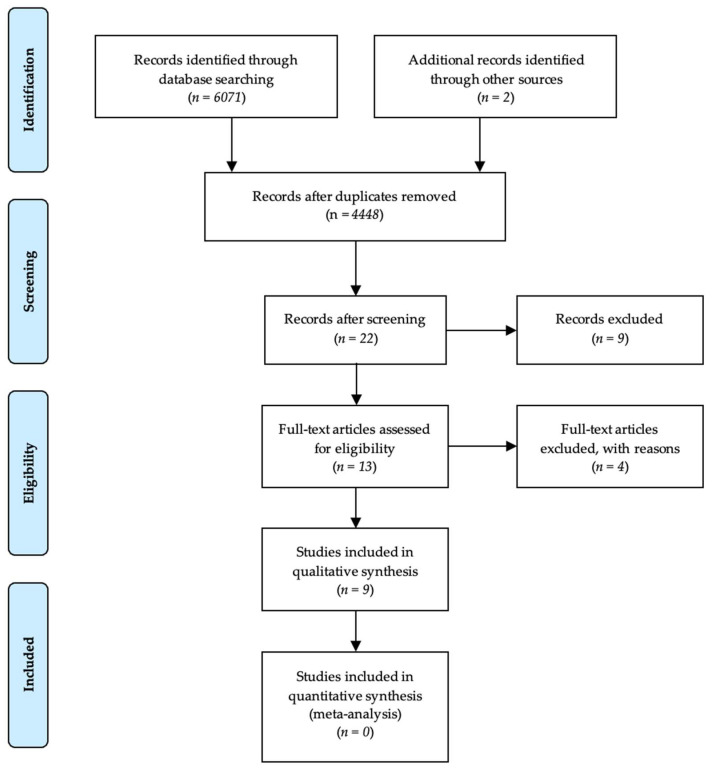
PRISMA flow chart—study selection procedure.

The procedure for the study selection in this systematic review is presented in Figure 1.

The bibliographic research in PubMed/Medline, Scopus, and Web of Science databases resulted in 6071 results. This research was supplemented by research in previously published systematic reviews, which identified two additional studies. After excluding non-relevant studies, a total of nine original articles were included. 

The analysis of the included studies regarding the type of study, study design, number of patients, age of patients, type of investigated glioma, stage of the disease, type of interventions performed, follow-up time, and type and duration of the KD is presented in Table 1. 

Table 2 presents the detailed data regarding patient survival, symptoms, and quality of life.

### 3.1. General Characteristics of Studies

The studies included in this review were published between 2014 and 2021, and more recent data were included in this systematic review in comparison with previous reviews.

Of the nine included studies, six (66.7%) were prospective studies and two (22.2%) had a control group. In most studies, it was found that the number of included patients was low, with an average of 8.3 (3 to 17) patients per study. 

The age of patients included in the studies ranged from 5.3 years old to 72 years old. Two studies were conducted in children between 2.5 and 15 years old, and 5.3 and 15.5 years old. The remaining studies were performed on adults aged between 45 and 72 years old. The overall mean age of patients with gliomas included in the analyzed studies was 42.1 years old, with a standard deviation of 19.9 years old.

The time of dietary intervention ranged from three weeks to 26 months. The follow-up period ranged from 12 weeks to 26 months.

### 3.2. Characteristics of Gliomas

Concerning the type of investigated glioma, the studies observed elevated stage gliomas such as glioblastomas in 66.7% of the studies, astrocytomas in 22.2% of the studies, intrinsic pontine gliomas in 22.2% of the studies, and glioblastomas multiforme in 11.1% of the studies. Grade II (22.2%) and grade III (33.3%) gliomas were included, and patients with grade IV glioma were present in all studies. 

Regarding the treatment of gliomas, in these studies, all patients underwent joint radiotherapy with temozolomide. However, these were not the only interventions applied. Drugs such as temozolomide, gemcitabine, prednisolone, bevacizumab, and lomustine were applied individually in four different studies (33.3%): temozolomide in the studies by van der Louw et al., 2019b [15] and Perez et al. [27]; bevacizumab and lomustine in the study by Rieger et al. [8]; lomustine in the study by Martin-McGill et al., 2018 [17]; and gemcitabine and prednisolone in the study by van der Louw 2019a [10]. In one study, patients received chemotherapy and concomitant radiotherapy as therapeutic interventions since the initial treatment with radiotherapy had no effect [28].

### 3.3. Features of the Implemented Diet

In the analyzed studies, we verified the implementation of different types of KD.

In the study by Rieger et al., KD restricted carbohydrate intake to 60 g per day, including intake of 500 mL of highly fermented yogurt drinks and two vegetable oils. No energy restriction was applied [8].

Martin-McGill et al., 2018 applied a Modified Ketogenic Diet (MKD) comprising 70% dietary lipids, while carbohydrates were limited to 20 g per day, corresponding to 3–5% of TEV. The protein content had no restriction [17].

In the study by van der Louw et al., 2019b, KD included two phases. During the initial eight weeks, associated with radiotherapy and temozolomide, KD was exclusively liquid with a ratio of 4:1. Over the next six weeks, KD transitioned to solid foods at a ratio of 1.5–2.0:1, with MCT [15].

Champ et al. restricted carbohydrate intake to <50 g per day, corresponding to 8% of TEV. Lipids accounted for 77% of the energy value and proteins 15% [16].

In the study by van der Louw et al. 2019a, patients started KD with a commercial liquid enteral formula at a ratio of 4:1 for a maximum of two weeks. When ketone levels reached 3 mmol/L, the KD was changed to a ratio of 1.5:1–2.0:11. During the study, MCTs were administered with KD [10].

In the study by Klein et al., the authors applied a KD in a ratio of 4:1 in two different groups. In group 1 included patients with recent glioblastoma; in group 2, patients with recurrent glioblastoma were included. The diet included 1600 kcal per day, with a total of 10 g of carbohydrates [29].

Perez et al. grouped patients undergoing various types of KD, including KD with MCT, CKD, and MAD. However, this study did not specify the proportions or amounts of macronutrients applied [27].

Martin-McGill et al. 2020 divided patients into two groups with the aim of implementing two different KDs: group 1 corresponded to a KD with MCT, and group 2 to an MKD. However, as in the study by Perez et al., no proportion or amount of administered macronutrients were reported [30].

In the study by Panhans et al., a 3:1 ratio KD was applied. The intake of carbohydrates was limited to ≤20 g per day [28].

Although the analyzed studies implement different types of KD, we can verify that the studies by Martin-McGill et al., 2018, Klein et al., and Panhans et al. applied more restrictive diets, having a maximum carbohydrate intake of 20 g per day. On the other hand, the studies by Rieger et al. and Champ et al. implemented more liberal KDs with daily carbohydrate intakes of ≤60 g and 50 g, respectively.

### 3.4. Overall Patient Survival

Patient survival varied from study to study. The study by Martin-McGill 2018 et al. made no reference to patient survival.

The mean of maximum overall survival was 25.4 months in the study by Klein et al., while the lower overall survival corresponded to the study by Rieger et al., with a median of 32 weeks (≈8 months) of survival [8,29].

Rieger et al. implemented KD for 16 weeks, where ketosis was reached and kept stable in most patients. There was stabilization of the disease at six weeks of diet, with this stabilization being balanced in an average duration of 12 weeks. The median overall survival after starting the diet was 32 weeks (≈8 months), ranging between 6 weeks (≈1.5 months) and 86 weeks (≈21.5 months) [8].

Overall survival in the study by Champ et al. had an average of 14 months, and the recurrence of the disease was, on average, 10.3 months. KD was implemented throughout the follow-up period (14 months) [16].

In the study by van der Louw et al. 2019a, the KD implementation lasted three months. In this study, diffuse intrinsic pontine gliomas that have a median overall survival of 9 to 11 months were evaluated [10]. The overall survival times observed in this study were 6.4, 16.5, and 18.7 months for each of the participants. The 6.4-month survival was explained by a severe decrease in consciousness after a generalized tonic–clonic crisis in one of the patients, who had only completed three weeks of the dietary intervention. Survival after starting nutritional therapy was 6.5 months for the remaining cases [10].

In the study by van der Louw et al. 2019b, overall survival averaged 12.8 months, with two patients achieving survival times of 17.7 and 19 months. Ketogenic therapy was performed for 14 weeks, and patients reached ketosis after an average of 4.5 days [15].

The study by Klein et al. was divided into two groups. Both groups received KD as therapy, with group 1 including patients with recent glioblastoma and group 2 including patients with recurrent glioblastoma. KD was implemented for six months. The mean survival from the onset of KD was 20 months for group 1 and 12.8 months for group 2. However, in terms of overall survival, group 1 had an average of 21.8 months and group 2 had an average of 25.4 months [29].

**Table 1:** Overview of studies regarding their design, subjects, type of investigated glioma, and therapeutic interventions.

Study	Type of Study	Study Design	N	Age of Patients	Type of Glioma	Disease Stage	Interventions	KD Duration	Follow-up Time	Type of KD
Rieger 2014 [8]	Controlled	Prospective	17	Med 57 years (30–72)	Glioblastoma	Grade IV	RT + Temozolomide; Bevacizumab and Lomustine	3 to 16 weeks	16 weeks	Max. 60 g carbs/day; highly fermented yogurt drinks (500 ml per day) and two different vegetable oils (base oil and addition oil)
Champ 2014 [16]	Not controlled	Retrospective	6	Med 54 years (34–62)	Glioblastoma	Grade III–IV	RT + Temozolomide	14 months	14 months	KD ≤ 50 g carbs/day
Martin-McGill 2018 [17]	Controlled	Prospective	6	Med 46 years (34–49)	Glioblastoma Anaplastic astrocytoma	Grade II–IV	RT + Temozolomide; Lomustine	12 weeks	12 weeks	Max. 20 g carbs/day, 70% lipids
van der Louw 2019a [10]	Not controlled	Prospective	3	Med 11.6 years (5.3–15.5)	Diffuse intrinsic pontine glioma	Grade IV	RT + Temozolomide; Gemcitabine; Prednisolone; Temozolomide	3 months	3 months	Liquid KD: 4:1 (+MCT)
van der Louw 2019b [15]	Not controlled	Prospective	9	Med 53.8 years (33.5–65.5)	Glioblastoma	Grade IV	RT + Temozolomide	14 weeks	14 weeks	Liquid KD 4:1 (8 weeks): 11 g carbs solid KD + MCT 1.5-2:1 (6 weeks): 57 g carbs
Klein 2020 [29]	Not controlled	Prospective	5	Med 49.8 years (40–64)	Glioblastoma	Grade IV	RT + Temozolomide	6 to 26 months	26 months	KD 4:1; 10 g carbs/day
Martin-McGill 2020 [30]	Controlled	Prospective	12	Med 57 years (44–66)	Glioblastoma	Grade IV	RT + Temozolomide	38 days to 12 months	12 months	KD with MCT/Modified KD
Panhans 2020 [28]	Not controlled	Retrospective	12	Med 45 years (32–62)	Glioblastoma multiforme, astrocytoma, oligodendroglioma	Grade II–IV	Chemotherapy + RT; RT; RT + Temozolomide	120 days	120 days	KD 3:1; ≤ 20 g carbs/day
Perez 2021 [27]	Not controlled	Retrospective	5	Med 4.4 years (2.5–15)	Diffuse intrinsic pontine glioma	Grade IV	RT + Temozolomide; Chemotherapy HIT-SKK; Temozolomide	6.5 months (0.25 to 2 years)	2 years	Classic KD/KD with MCT/Modified Atkins Diet

RT, radiotherapy; Med, median; MCT, medium-chain triglycerides; carbs, carbohydrates; N, number of patients; KD, ketogenic diet; Max., maximum; HIT-SKK, Therapieprotokoll für Säuglinge und Kleinkinder mit Hirntumoren (Therapy protocol for infants and young children with brain tumors).

**Table 2:** Overview of study results on survival, symptoms, and quality of life.

Study	Patient Survival	Symptomatology Associated with Dietary Intervention	Quality of Life
Rieger 2014 [8]	Med 32 weeks (between 6 and 86 weeks).	Weight loss, diarrhea, constipation, hunger	Decreased quality of life
Champ 2014 [16]	Med 14 months	Constipation, asthenia, weight loss, nephrolithiasis, hypoglycemia	Not available
Martin-McGill 2018 [17]	Not available	Constipation	Improved quality of life
van der Louw 2019a [10]	Between 16.5 and 18.7 months	Hypoglycemia, hyperketosis, vomiting, refusal to eat, asthenia, constipation	Decreased quality of life
van der Louw 2019b [15]	Between 9.8 and 19.0 months (Med 12.8 months)	Constipation, nausea/vomiting, hypercholesterolemia, hypoglycemia, diarrhea, low carnitine concentration	Decreased quality of life
Klein 2020 [29]	Group 1: $\bar{x}$ =21.9 months (between 11 and 29.2 months) Group 2: $\bar{x}$ =25.4 months (between 13.9 and 38.7 months)	Weight loss, hunger, nausea, dizziness, asthenia, constipation	Improved quality of life
Martin-McGill 2020 [30]	Med 67.3 weeks	Hypokalemia, hypocalcemia, hypernatremia, hyperkalemia, constipation	Improved quality of life
Panhans 2020 [28]	Between 9.8 and 19.0 months	Asthenia, weight loss, nausea, vomiting, headache, decreased appetite	Improved quality of life
Perez 2021 [27]	Med 18.7 months	Hypoglycemia, constipation, hyperketosis, vomiting, asthenia, hyperuricemia	Not available

Med, median; $\bar{x}$, mean.

Martin-McGill et al., 2020 divided the patients into two groups, distinguishing the implemented KDs. Group 1 corresponded to a KD with MCT and group 2 to an MKD. In both groups, ketosis was reached within the first six weeks. Overall survival had a median of 67.3 weeks (≈16.8 months) [30].

Panhans et al. applied KD for at least 120 days. Ketosis was achieved in most patients within the first week of therapy. Overall survival ranged from 9.8 to 19 months [28].

The study by Perez et al. included patients who had implemented ketogenic therapy for a period longer than three months. The estimated overall survival was 18.7 months. There were also two patients who achieved survival times of 22 and 30 months [27].

The study by Martin-McGill et al., 2018 did not report patient survival [17,31]. However, the KD was implemented for 12 weeks, and three patients continued this therapy for more than 360 days [17], which is a possible assumption regarding survival in this study. 

### 3.5. Associated Symptomatology

The symptoms associated with KD intervention detailed in the studies included 75 patients and are shown in Figure 1. 

**Figure 2:**
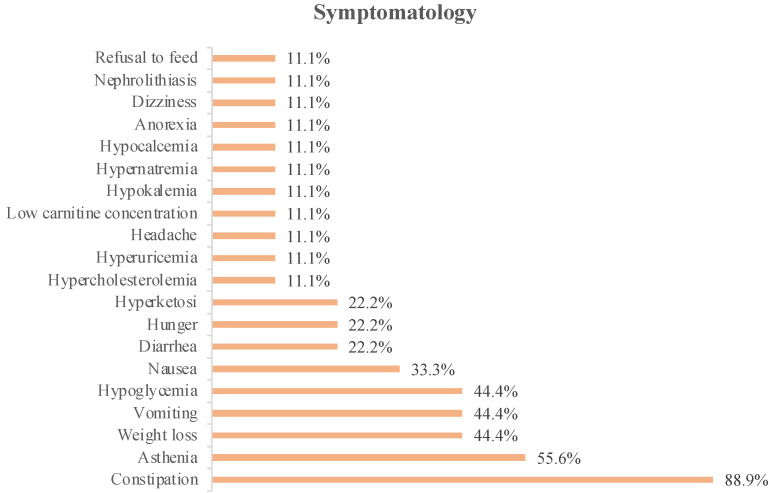
Symptomatology associated with KD therapy in glioma patients.

In Figure 2, we can see that constipation was the most reported symptom in the analyzed studies (88.9%). This was followed by asthenia, which was reported in 55.6% of the studies.

In 44.4% of the studies, weight loss, vomiting, or hypoglycemia were reported as associated symptoms, whereas in 33.3% of the studies, nausea was reported.

Patients reported diarrhea, hunger, or hyperketosis as associated symptoms in 22.2% of the analyzed studies, and only 11.1% of the studies reported dizziness, anorexia, food refusal, hypernatremia, hypokalemia, hypocalcemia, low carnitine concentration, headache, hyperuricemia, or hypercholesterolemia.

Important symptoms associated with KD include hypoglycemia, weight loss, vomiting, diarrhea, and nausea. In this review, hypoglycemia was reported in the studies by Champ et al., van der Louw et al., 2019a and 2019b, and Perez et al. Hypoglycemia, reported in the study by Champ et al., was asymptomatic [16]. Weight loss was reported in studies by Rieger et al. [8], Champ et al. [16], Klein et al. [29], and Panhans et al. [28]. Vomiting was reported in the two studies by van der Louw et al. [10,15], in the study by Panhans et al. [28], and in that of Perez et al. [27]. Nausea was reported in the studies by van der Louw et al., 2019b [15], Klein et al. [29], and Panhans et al. [28]. Diarrhea was referenced in the studies by Rieger et al. [8] and van der Louw et al., 2019b [15]. The authors referred to weight loss as a minimal side effect [8,16,17].

It should be noted that in the study by van der Louw et al., 2019b, patients with dexamethasone were excluded [15], while in the studies by Champ et al., Rieger et al., Martin-McGill et al., 2018, Panhans et al., Klein et al., and Martin-McGill et al., this exclusion was not made, which could influence the symptomatology and the interpretation of blood glucose values [8,16,17,28,29,30].

Only one study reported the occurrence of hypercholesterolemia [10]. The remaining studies did not report changes in the lipid profile.

### 3.6. Quality of Life 

Quality of life is a parameter that must be evaluated with appropriate objective or subjective measures, with the implementation of a validated questionnaire, for example. Only three studies applied quality of life perception questionnaires [8,15,30]. The remaining studies presented subjective results.

**Figure 3:**
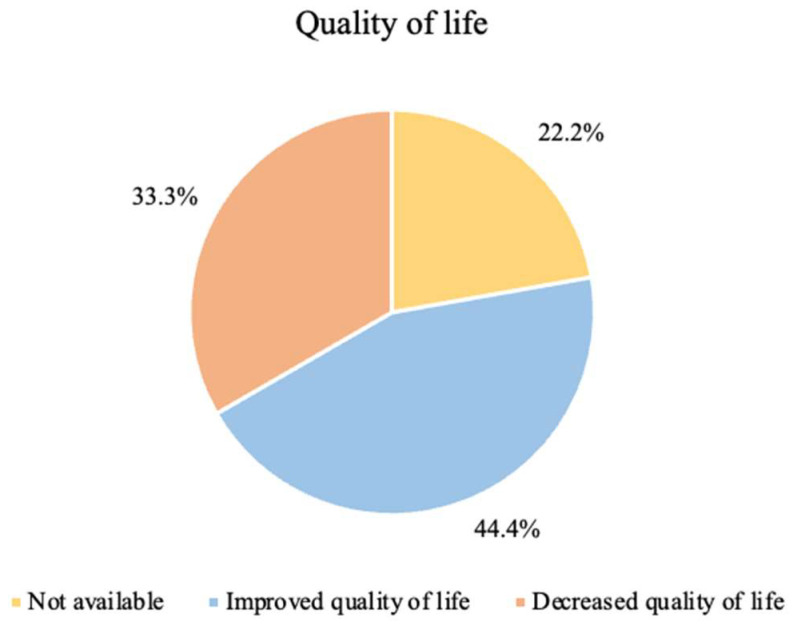
Perception of quality of life in glioma patients undergoing KD.

The perception of quality of life felt by patients with the implementation of ketogenic therapy is shown in Figure 3. 

As previously stated, not all studies analyzed the quality of life of patients undergoing treatment—22.2% did not report any results on quality of life, since they were not indicators studied by the authors themselves.

In 44.4% of the studies, there was an improvement in the quality of life with the implementation of KD, regardless of the symptoms felt.

In 33.3% of the studies, there was a decrease in the quality of life. In two of these studies (Rieger et al. and van der Louw et al., 2019b), the authors estimated that this decrease was related to the symptoms experienced throughout the studies and to tumor progression [8,15].

Martin-McGill et al., 2020 reported that patients who withdrew from KD therapy justified it with a decrease in quality of life in comparison to the beginning of treatment. However, patients who remained on therapy reported an improvement in quality of life, as they had a sense of control during tumor treatment [30].

Studies that did not objectively assess the quality of life assumed a good quality of life or an improvement in quality of life. Martin-McGill et al., 2018 reported a good quality of life for patients, with an improvement in symptoms [17].

The study by Panhans et al. mentioned that a qualitative assessment of the quality of life was carried out, in which there was an improvement in energy, mood, neurocognitive function, general well-being, and symptoms [28].

Klein et al. did not assess the quality of life. Nonetheless, they referred to a patient who improved his quality of life. It should be noted, however, that in this study, the meals were prepared by a company and provided free of charge to patients [29].

In the study by van der Louw et al., 2019a, it was mentioned that KD was not compatible with a better quality of life for patients [10], which led to the understanding that with the implementation of KD, there was a decrease in the quality of life. In this study, the children were in palliative care, and hospitalization of two children was necessary for the management of epileptic seizures and implementation of enteral nutritional support through a nasogastric tube due to swallowing difficulties. These data were not related to the implementation of KD, however, with direct influence in the perceived quality of life [10].

The studies by Champ et al. and Perez et al. did not make any reference to the patients’ perception of quality of life during or after treatment with implementation of KD [16,27,32]. Even so, in the study by Perez et al., it was mentioned that patients had an improvement in symptoms [27].

### 3.7. Bias Risk Analysis 

**Figure 4:**
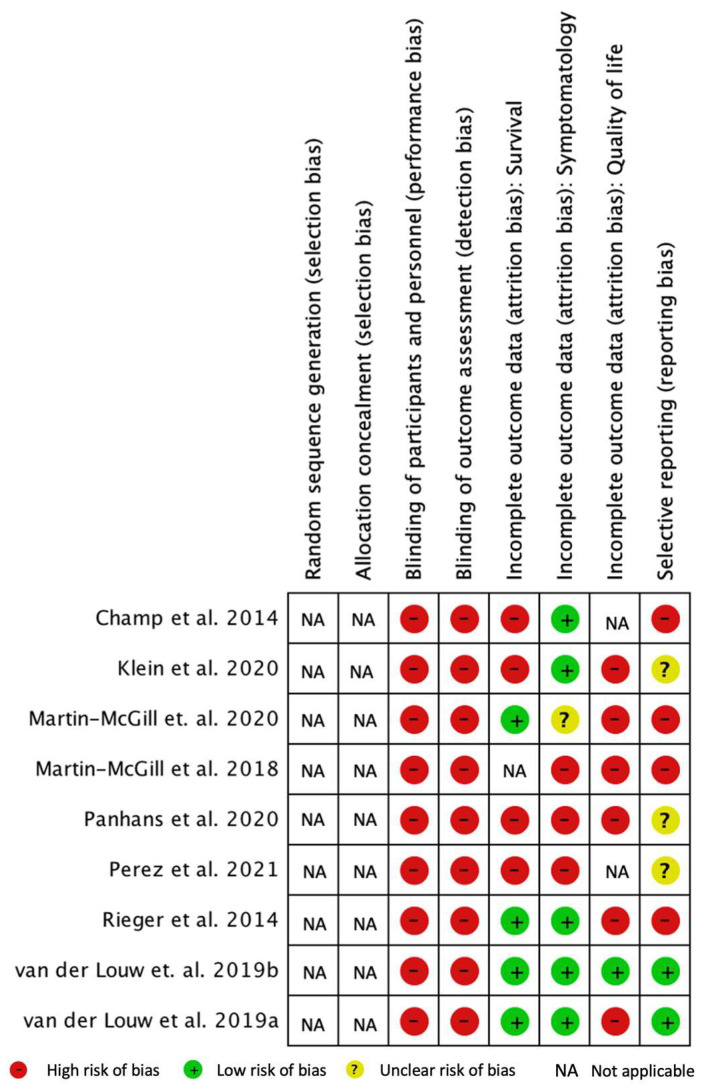
Risk of bias summary—risk of bias analysis for each included study [8,10,15,16,17,27,28,29,30].

The results of the quality assessment of the clinical trials are shown in Figure 4. To assess the risk of bias, Cochrane RevMan 5 software (Review Manager 5.4.1) [26] was used.

The performance bias includes the knowledge of the interventions by the participants and the team that is part of the study. Due to uncontrolled studies, there was no blinding, which led to a high risk of performance bias.

The detection bias refers to the concealment of the investigators about the therapeutic interventions in order not to influence the results. There was a high risk of detection bias because the authors were not blinded to the diet therapy.

Attrition bias encompasses the tendency to attrition due to the quantity, nature, or manipulation of incomplete result data. Data on patient survival (essential variable), symptomatology, and quality of life (secondary variables) were evaluated.

A high risk of attrition bias due to incomplete assessment of the quality of life outcomes was notable in most studies. Only the study by van der Louw et al. 2019b presented a low risk of bias [15], being the only study that applied a quality of life questionnaire and presented those results.

We verified a low risk of attrition bias regarding the nature of the data and the evaluation of the results for survival in four studies [8,10,15,30]. In the remaining four studies, there was a high risk of bias [16,27,28,29] since it was observed that survival was reported by observation of patients or through qualitative data, so there was no application of statistical tests.

Regarding symptoms, we found a low risk of attrition bias for most studies [8,10,15,16,29]. However, three studies showed a high risk of bias [17,27,28]: the study by Martin-McGill et al., 2018 and by Panhans et al., due to the reporting of symptoms (no questionnaire or structured interview method was applied), and in the study by Perez et al., as results were presented for only three patients, with incomplete data. The study by Martin-McGill et al. 2020 presented an uncertain risk of bias [30] and reported that they evaluated ketosis, food acceptability, and gastrointestinal adverse events and, later, also presented mineral deficits.

Reporting bias considers bias due to the description of selective results. The studies by Champ et al., Klein et al., Martin-McGill et al., 2018, Martin-McGill et al., 2020, and Rieger et al. were at high risk of bias [8,16,17,29,30]. The studies by van der Louw et al., 2019a and 2019b had a low risk of bias [10,15].

## 4. Discussion

Evidence for the use of KD in clinical practice is still very limited. There has been an attempt to further investigate this topic, which implies new studies to be implemented in the search for more reliable, promising, and relevant results.

The main objective of the present systematic review was to analyze and systematize the clinical studies that tested KD in the context of glioma treatments, focusing the analysis of the evidence on the potential therapeutic value of KD as a treatment option in patient survival, and to further evaluate the potential effects of KD on associated symptoms and quality of life of these patients. A total of nine original studies that reported at least one of the study objectives were identified and analyzed.

It should be noted that, of the nine studies included, two were applied to children between 2.5 and 15 years old and 5.3 and 15.5 years old. The remaining studies were applied to adults aged between 45 and 72 years old.

### 4.1. Overall Patient Survival 

KD, being a therapy that reduces blood glucose levels and increases ketone body levels, can be an effective therapy to increase the survival of patients with gliomas [32].

In the studies included in this review, we found that different results were described, with survival values between 32 weeks (approximately eight months) and 25.4 months. Survival is a factor that may vary mainly with age, disease stage, adherence to therapy, treatment efficacy, and tumor location. In this review, the mean overall survival was found to be around 15.9 months.

The survival interval of patients with gliomas on common therapy is between 12 and 15 months [13]. With the implementation of KD, it was possible to verify that survival in the studies by Champ et al. and van der Louw et al. 2019b corresponded to this interval [16], with no improvement in comparison to the survival time considered in the literature. Patients included in the studies by van der Louw et al., 2019a, Klein et al., Martin-McGill et al., 2020, and Perez et al. exceeded the average survival of those undergoing common therapy [10,27,30]. The study by Rieger et al. was the only one in which the survival time was lower than the average time of patients with gliomas undergoing common therapy [8]. According to the authors, the reasons for the low clinical activity may be due to the failure to significantly reduce glucose through KD or to the hypothesis that tumor cells bypassed the glucose reduction using ketone bodies [8].

The study that showed a longer survival was reported by Klein et al., where a KD with a ratio of 4:1 was implemented, with 1600 kcal per day and a total of 10 g of carbohydrates [29]. On the other hand, patients from Rieger et al., who implemented a KD without energy restriction but with restriction of carbohydrate intake to 60 g per day, had the lowest survival [8].

In this analysis, the type of KD that presented the highest overall survival was CKD, with a ratio of 4:1, verified in the studies by Klein et al. and van der Louw et al. 2019b. Although the studies by Martin-McGill et al. and Perez et al. showed an increase in overall survival, they applied different types of KD, with no description of the proportions of macronutrients provided.

In most of the studies analyzed with the implementation of KD therapy, the overall survival exceeded the prognosis of patients with the usual chemotherapy and/or radiotherapy associated with temozolomide. A previously published review also mentions that KD improved the survival of most patients [33].

In this systematic review, all studies included grade IV gliomas, two studies included grade II gliomas, and one study included a grade III glioma. The overall survival data shown in the included studies are not presented according to glioma type. As such, it is not possible to assess if this variable is affected by the glioma type. 

As gliomas have different levels of malignancy, it is expected that the type of tumor can impact overall survival. Further analysis of this matter is recommended to fully understand how overall survival varies according to malignancy. Therefore, future large-scale perspective studies including a greater variety of glioma types are needed.

### 4.2. Associated Symptomatology 

The symptoms associated with KD may involve gastrointestinal problems such as vomiting, diarrhea, constipation, gastroesophageal reflux, and others such as hypoglycemia, dizziness, asthenia, hyperketonemia, or metabolic acidosis. Other complications may also arise, such as dyslipidemia, with hyperlipidemia, hypercholesterolemia, and hypertriglyceridemia; cardiovascular diseases, such as cardiomyopathy; nephrolithiasis, and vitamin and mineral deficits [34,35,36,37].

The symptomatology associated with the therapy, or the adverse effects felt by the patients, were consistent in most of the studies, and the symptom most reported by the patients was constipation [8,15,16,17,27,29,30]. Asthenia was the second most mentioned symptom, being described in studies by Champ et al. [16], Klein et al. [29], Panhans et al. [28], van der Louw et al., 2019a [10], and Perez et al. [27].

The most expected adverse effect in KD is hypoglycemia, due to the low concentrations of carbohydrates and the amount of energy provided in the diet [10,15,16,27]. In this review, hypoglycemia was found in four studies, but it promptly resolved with a change in dietary and medical therapy. Other important symptoms associated with all types of KD were weight loss, vomiting, diarrhea, and nausea.

Although these symptoms were described during dietary therapy, they were considered to have a mild and transitory incidence. Some authors considered that there was no severe symptomatology associated with KD and related the occurrence of serious side effects to the disease itself and/or medical treatment [15,27,31].

According to the literature, the KD type that has the most side effects is CKD. However, in this review, it was possible to verify that the symptoms felt by the patients were transversal, regardless of the diet implemented. Thus, side effects should not be a reason to select a less restrictive diet.

### 4.3. Quality of Life 

Quality of life is a parameter that must be evaluated with the appropriate objective and subjective measures to obtain reliable results [38]. With the implementation of quality of life questionnaires, it would be possible to assess this parameter with some certainty.

In this review, we were able to verify that only three studies applied quality of life questionnaires, namely, Rieger et al., Martin-McGill et al., 2020, and van der Louw et al., 2019b [8,15,30], and the only validated questionnaires were those used by Martin-McGill et al., 2020. Although the authors applied these questionnaires, only the study by van der Louw et al. 2019b presented the results obtained, while Rieger et al. reported qualitative results and Martin-McGill et al., 2020 mentioned global health status.

The association between the symptoms felt by the patients and their quality of life was notable. This is supported by Rieger et al., van der Louw et al., 2019b, and Martin-McGill et al., 2018, who associated a decrease or improvement in quality of life with an increase or improvement in symptoms, respectively.

In this systematic review, quality of life was a variable related to a high risk of attrition bias. Therefore, we recommend that other variables should be considered in future interventions, as these represent a directly dependent factor on the stage of the disease, general well-being, symptoms, and the patient’s response to therapy. With the results obtained, it became difficult to establish a relationship between quality of life and the implementation of KD, so we could only see its inconsistency.

### 4.4. Study Limitations 

This systematic review applied a consistent methodology, with well-defined inclusion and exclusion criteria, and covering different databases. All studies published to date were included.

The analysis of articles for the selection of relevant and quality publications was carried out by two reviewers, reducing the risk of errors in the selection of studies. In cases of doubt or contradictory opinion, a third element was involved in the selection of studies to be included.

The present systematic review followed the PRISMA recommendations and was published in PROSPERO, with the identification CRD42021264173. Cochrane RevMan 5 software, indicated for the development of systematic reviews, was used to analyze the risk of bias of the included studies. This made it possible to analyze and evaluate these studies in depth.

Although there is a recent publication that included most of the studies here analyzed, in the present study, a recent 2021 study (Perez et al.) [27] was also analyzed, including these data in a systematic review for the first time. Additionally, our review is the only one to date that includes the analysis of side effects, survival, and quality of life.

## 5. Conclusions

Some limitations were found in the quality of the analyzed data, including the sample size in the studies (between three and seventeen patients), the different types of gliomas, and the absence of a control group in most studies. This lack of control makes it difficult to establish a solid conclusion on the efficacy of KD in the treatment of gliomas. 

Since patients must be informed and accept the dietary modifications that KD requires, performance bias or even the placebo effect could be present. Although the results are made more robust when interventions are hidden, in the implementation of this kind of therapy, this is a challenge. 

However, in most studies analyzed with the implementation of KD therapy, overall survival exceeded the prognosis of these patients with the usual chemotherapy therapy and/or radiotherapy associated with temozolomide. Both the use of different types of KD, and the presentation of data as a report and not in a more structured way, made it difficult to compare the side effects felt by patients during the implementation of the different types of diets. 

Furthermore, in the assessment of the quality of life, the use of reported data without the application of objective and validated measures constituted a limitation to the interpretation of the results. This represented a high risk of friction, and therefore, in most studies, the data collected were considered as having a high risk of bias. It was not possible to establish a relationship between quality of life and the implementation of KD. However, concerning overall survival, in most studies, the prognosis of patients with gliomas who implemented KD as therapy was higher compared to common therapy. 

Although the results obtained point to a positive effect of KD as adjuvant therapy of malignant gliomas, it is still important to develop new research of high quality that aims to minimize the risks of bias.

## Data Availability

The protocol for this systematic review was registered in the Inter-national Prospective Register of Systematic Reviews (PROSPERO) under the registration number CRD42021264173.
